# A rare case of supratentorial intraparenchymal ependymoma in a 13 years old girl: Diagnostic and therapeutic challenges

**DOI:** 10.12669/pjms.41.13(PINS-NNOS).13347

**Published:** 2025-12

**Authors:** Ahtesham Khizar, Haseeb Waheed, Mahroona Fatima Khalid, Haseeb Mehmood Qadri, Hassaan Zahid

**Affiliations:** 1 Ahtesham Khizar, MBBS, FCPS (Neurosurgery), Punjab Institute of Neurosciences, Lahore, Pakistan; 2 Haseeb Waheed, Medical Student Aga Khan University Hospital, Karachi, Pakistan; 3 Mahroona Fatima Khalid, MBBS, Postgraduate Resident Neurosurgery, Punjab Institute of Neurosciences, Lahore, Pakistan; 4 Haseeb Mehmood Qadri, MBBS, Postgraduate Resident Neurosurgery, Punjab Institute of Neurosciences, Lahore, Pakistan; 5 Hassaan Zahid, MBBS, FACS, FCPS (Neurosurgery), MS (Pediatric Neurosurgery), Punjab Institute of Neurosciences, Lahore, Pakistan

**Keywords:** Childhood ependymoma, Ependymoma, Neuroectodermal tumors, Pakistan, Supratentorial neoplasm

## Abstract

Ependymomas are neuroepithelial tumors that may arise from the ependymal cells of the cerebral ventricles, the central canal of the spinal cord, or cortical rests. This case report is of a 13 years old girl with no prior health issues who presented with symptoms of blurred vision, intermittent diplopia, tinnitus, vomiting, and vertigo that had worsened over three months. Except for these symptoms, her neurological examination was normal. Brain MRI revealed a large cystic tumor in the right parietal lobe, with enhancing nodules and surrounding edema that caused midline shift. The patient underwent surgical excision of the tumor that later came out to be ependymoma on histopathology. This case report aims to contribute to the medical literature by highlighting the rare occurrence of an ependymoma solely within the brain parenchyma. By sharing this unusual case, we hope to enhance understanding of this rare pathology and provide valuable insights regarding its occurrence in rare locations.

***List of Abbreviations:*** CE-CT: Contrast-enhanced CT; CE-MRI: Contrast-enhanced MRI; CNS: Central nervous system; CT: Computed tomography; GTR: Gross total resection; ICU: Intensive care unit; MRI: Magnetic resonance imaging; NCE-CT: Non-contrast enhanced CT, STR: Subtotal resection; WHO: World Health Organization.

## INTRODUCTION

Ependymomas are tumors that arise from the ependymal cells lining the ventricles in the central nervous system. These tumors are more prevalent in children, making up 2% of all childhood central nervous system (CNS) tumors, compared to 1.9% in adults.^1^ Males are slightly more likely to be affected than females. The location of ependymomas varies with age: in children, 90% of these tumors occur in the brain, whereas in adults, 65% occur in the spinal cord.^2^ Notably, about 70% of ependymomas develop in the lower part of the brain (infratentorial region), often originating from the fourth ventricle.^3^

Ependymomas typically arise in the ventricles and may extend into nearby brain tissue through the intima of the ependyma rather than originating solely in the brain parenchyma.^3^ Additionally, patient age is a crucial prognostic factor, with younger children facing a more challenging outcome.^4^ Adults tend to have a better outcome, with a 10-year overall survival rate of 79%. However, this rate drops to 28% for adults aged 75 and older.^1^ In contrast, children under three years of age have the poorest prognosis.^4^ Magnetic resonance imaging (MRI) is the preferred diagnostic tool for ependymomas, being the investigation of choice.^5^

Traditionally, treatment for ependymomas has involved surgery followed by radiation therapy. However, for very young children, chemotherapy is now being considered as an alternative to radiation, which can harm the developing brain.^6^ We present a rare and unusual case of cortical ependymoma in a young patient, a condition that is poorly understood in pediatric patients due to its extreme rarity, which will add to the available data and enhance knowledge regarding the entity. We present the following case in accordance with the CARE guidelines.^7^

## CASE PRESENTATION

A 13 years old girl, otherwise healthy, presented to the outpatient department of the Punjab Institute of Neurosciences, Lahore, Pakistan, in December 2024 with complaints of blurred vision, off and on diplopia, and tinnitus for three and a half months, associated with progressive vomiting and vertigo for two months. Symptoms were partially relieved with medications. The patient was developmentally normal, with no history of consanguinity, and family history was also not significant. She was conscious and well oriented in time, place, and person with normal behavior. She was neurologically intact, with no cranial nerve involvement and normal power, tone, and deep tendon reflexes.

**Fig.1:**
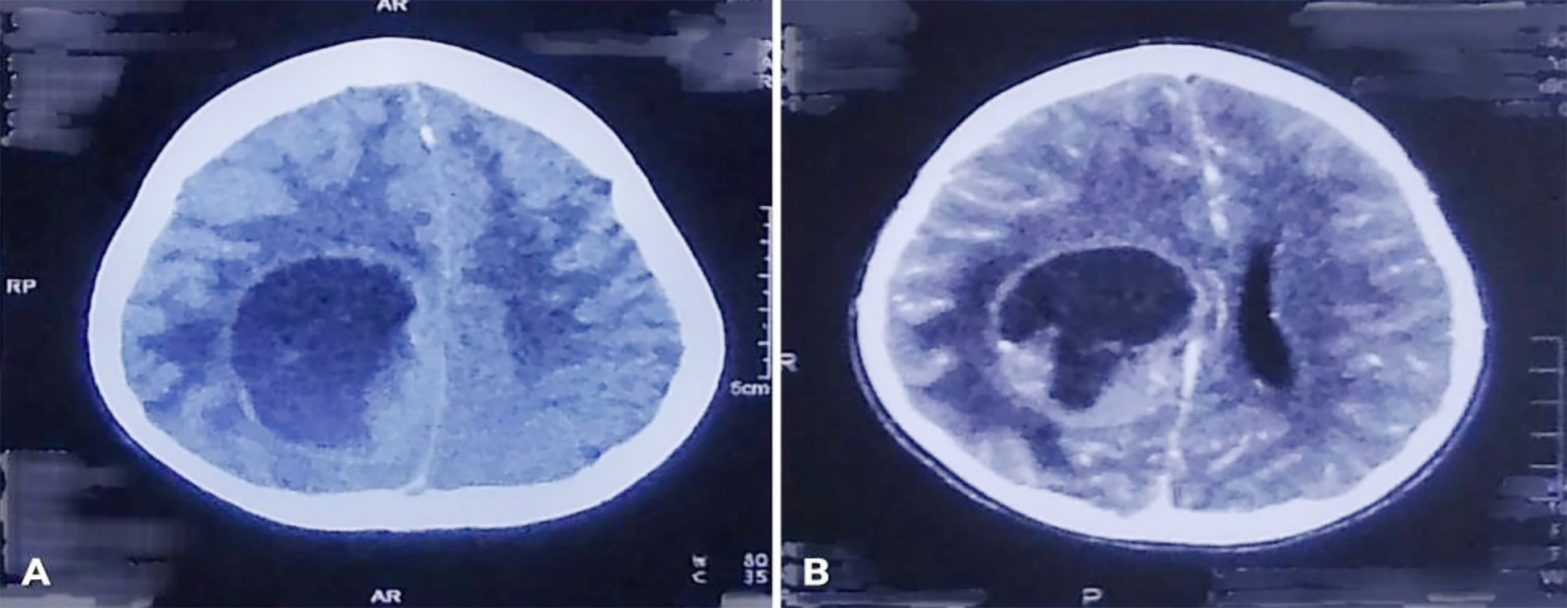
A: CT brain plain showing a well-defined, hypodense lesion in the right parietal region with perilesional edema and mass effect. B: CT brain with contrast showing a right-sided cystic lesion with an enhancing wall and mural nodule.

**Fig.2:**
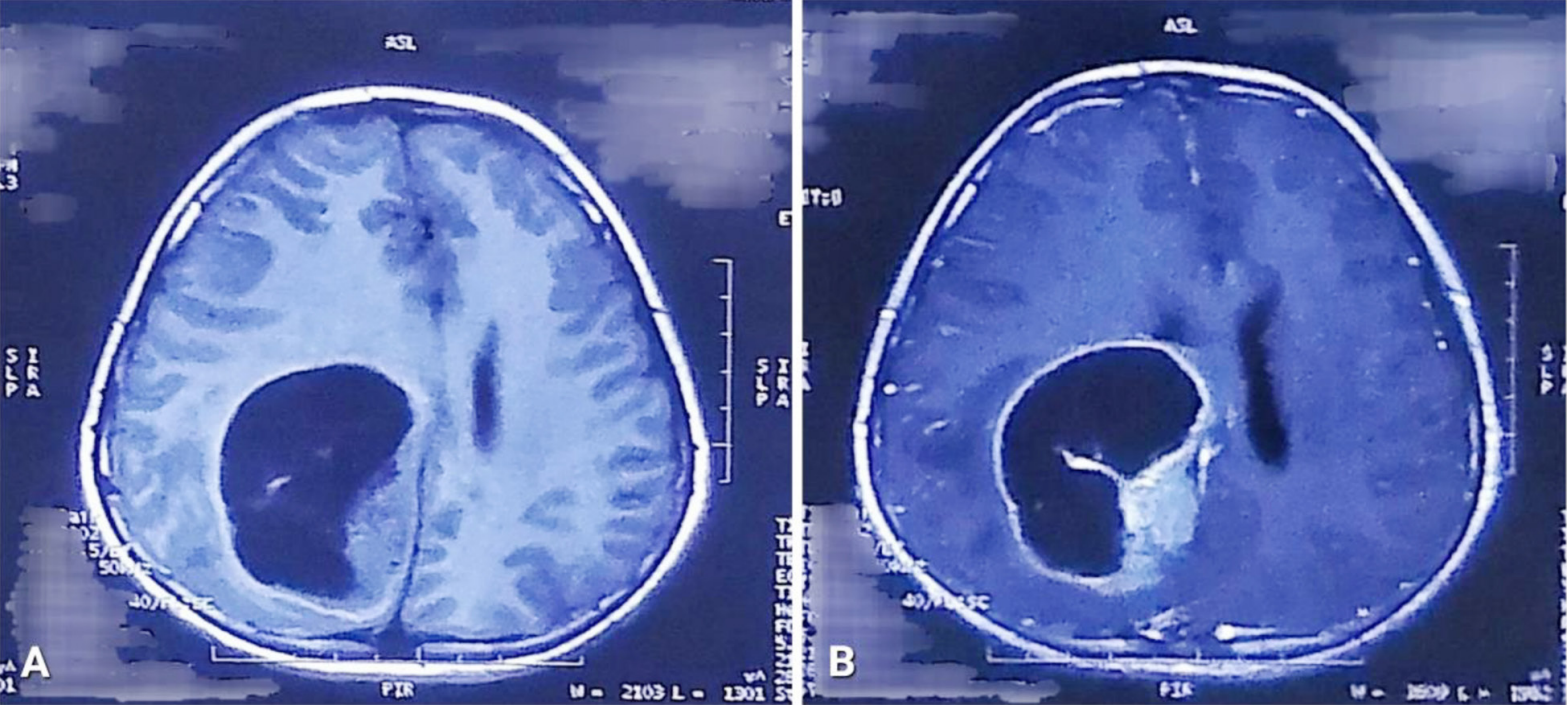
A: T1-weighted axial image showing a hypointense lesion in the right parietal region. B: T1-weighted contrast-enhanced axial image showing the contrast-enhancing wall of this cystic lesion with a peripherally located mural nodule.

**Fig.3:**
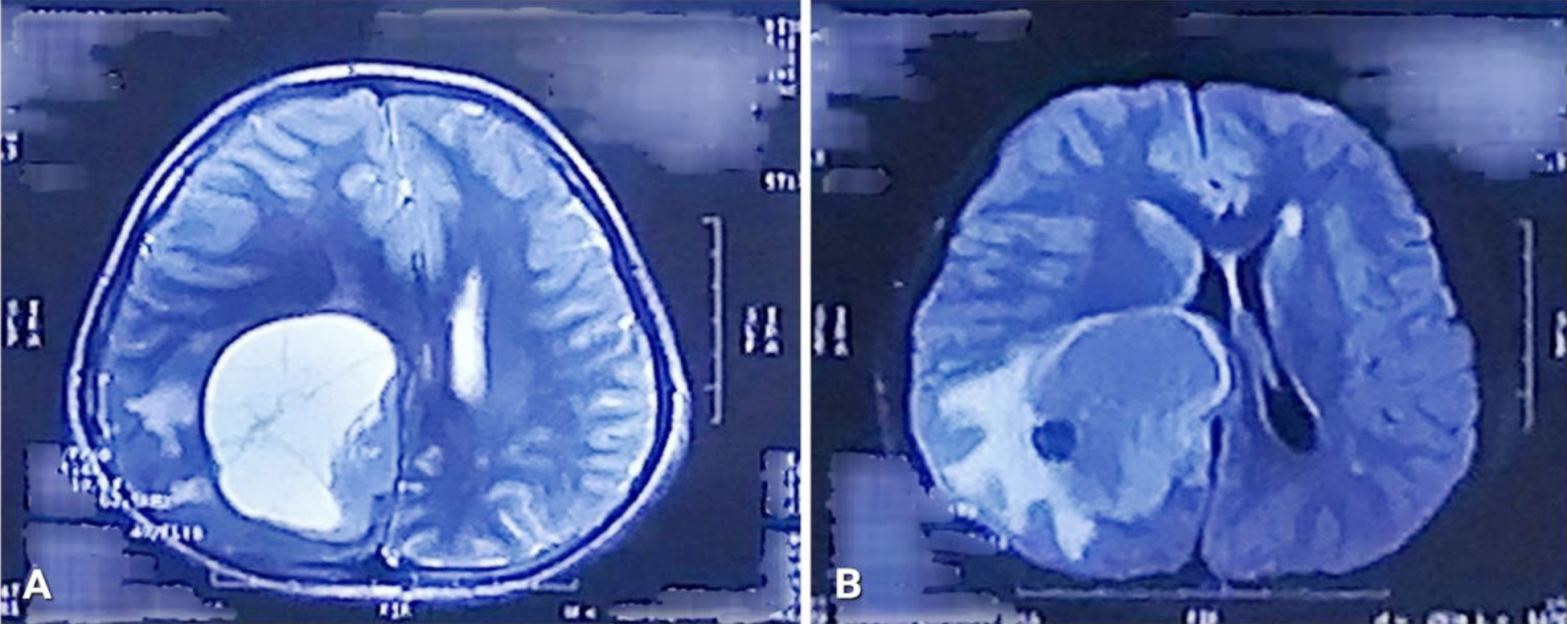
A: T2-weighted axial image showing a hyperintense lesion in the right parietal region. B: FLAIR image showing the same lesion with perilesional edema.

The patient was given a thorough diagnostic workup. Non-contrast enhanced computed tomography (NCE-CT) of the brain showed a well-defined, hypodense space-occupying lesion in the right parietal region with perilesional edema causing mass effect. (Fig.1A) Contrast-enhanced computed tomography (CE-CT) of the brain showed a right-sided cystic space-occupying lesion with an enhancing wall and mural nodule. (Fig.1B) Contrast-enhanced magnetic resonance imaging (CE-MRI) of the brain showed a 48 x 50 x 46 mm hypointense cystic mass lesion with contrast-enhancing peripherally located mural nodules in the right parietal lobe with adjacent perilesional edema causing a mass effect on the right lateral ventricle and midline shift. (Fig.2, 3, & 4) A pilocytic astrocytoma and pleomorphic xanthoastrocytoma were the differentials under consideration.

**Fig.4:**
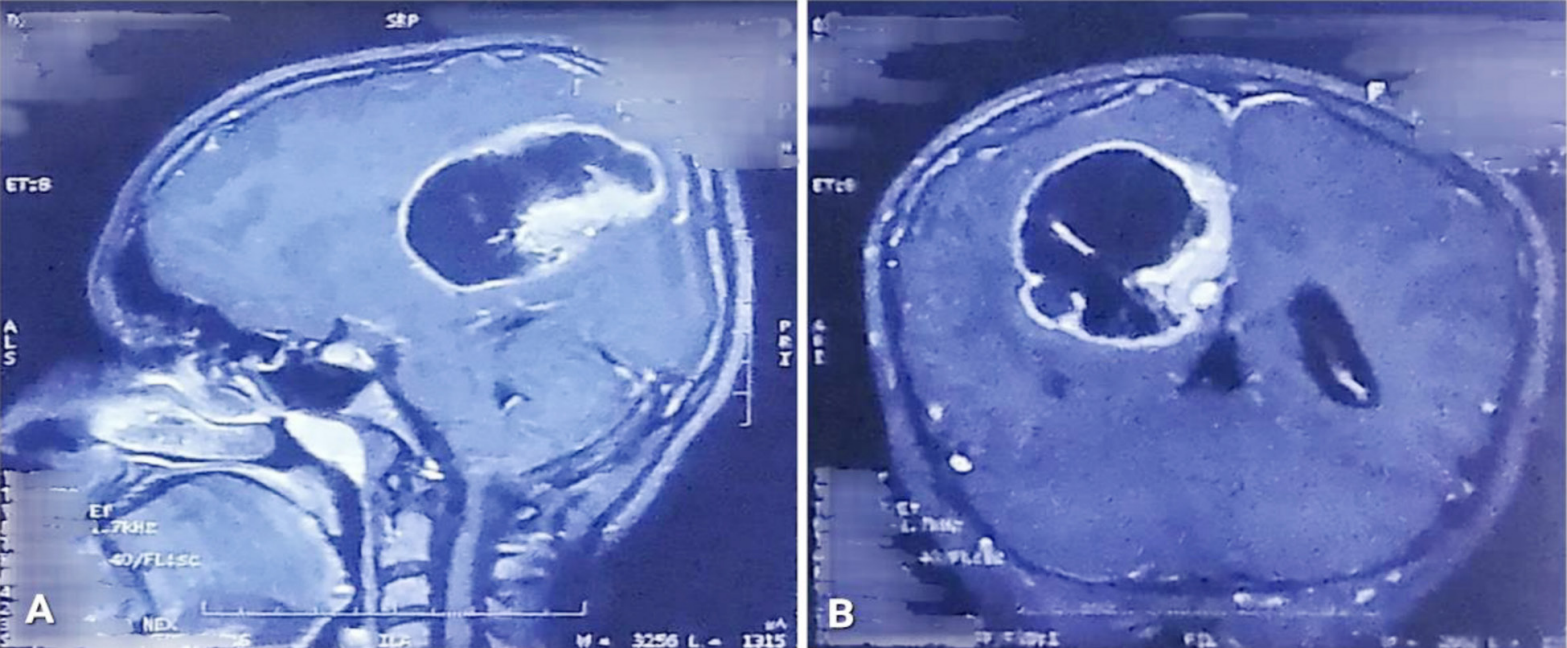
(A&B): T1-weighted sagittal and T1-weighted coronal contrast-enhanced images showing the same lesion with a contrast-enhancing wall and an enhancing mural nodule.

Surgical excision under general anesthesia was scheduled. The patient underwent right parietal lobe space-occupying lesion excision via quadrangular craniotomy. The lesion was excised completely with minimal blood loss. Intraoperative findings included 20 to 30 ml of yellowish cystic fluid with a grayish-colored solid portion adherent to the falx, moderately vascular, and hard in consistency. The surgery was smooth without any notable complications, and her emergence from general anesthesia was uneventful. Gross total resection (GTR) was achieved, and the immediate postoperative CT brain plain had no residual lesion or bleed. She was kept in the intensive care unit (ICU), under observation, for six hours, then shifted to the ward and discharged on the third postoperative day with no complications. At follow-up after two weeks, the patient had no new neurological deficits. There was no surgical site infection, and her attendants were reassured.

**Fig.5:**
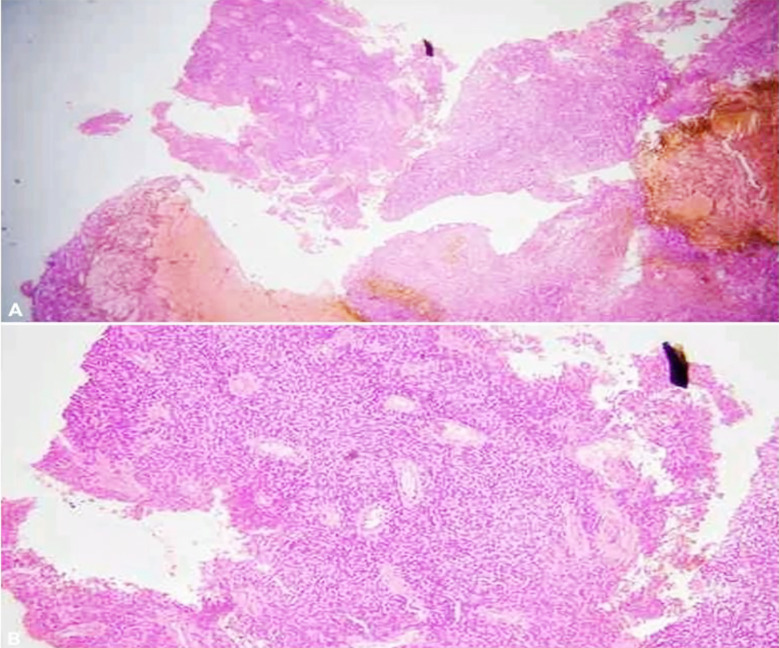
(A&B): On microscopy, sections reveal a neoplasm composed of round to ovoid cells having fine, evenly dispersed chromatin and fibrillary processes. The tumor cells are forming rosettes around small blood vessels (pseudorosettes) and around fibrillary matrices (true rosettes).

Histopathology finalized the diagnosis of World Health Organization (WHO) Grade-2 ependymoma, and a second opinion from another histo-pathologist was also taken. On microscopy, sections of the first laboratory revealed a neoplasm composed of round to ovoid cells having fine, evenly dispersed chromatin and fibrillary processes. The tumor cells were forming rosettes around small blood vessels (pseudorosettes) and around fibrillary matrices (true rosettes), as shown in Fig.5(A&B). Immunostains used were: EMA: positive, GFAP: positive, D240: positive, and OLIG-2: negative. Moreover, the sections of the second laboratory revealed a tumor composed of monomorphic round cells with spread chromatin. Perivascular pseudorosettes and gemistocyte-like cells were seen. The immunostains used were: STAT6: negative, OLIG-2: negative, GFAP: positive, and SSTR: negative.

At the one-month follow-up, her surgical incision had become infected, necessitating debridement and antibiotics. At the three month follow-up, CE-MRI revealed a 38 x 29 x 42 mm recurrent lesion that necessitated repeat surgery and postoperative radiation.

## DISCUSSION

Ependymomas, particularly those arising within the ventricular system, are well-recognized tumors of the central nervous system.^1^ However, as in our case, ependymomas that occur outside the ventricular system, particularly in an intraparenchymal location, are exceedingly rare and pose a unique diagnostic and therapeutic challenge owing to their slow-growing glial neoplastic nature.^8^ While these tumors are typically benign, they can be clinically challenging to distinguish from other glial tumors due to overlapping radiological and histopathological features.^9^ In these cases, accurate diagnosis often relies on detailed imaging, histopathology, and immunohistochemical analysis, given the similarities between ependymomas and other glial or neuronal-glial tumors. However, due to its silent behavior, its true incidence remains a mystery.^8^

Radiologically, ependymomas typically present as well-defined, contrast-enhancing lesions with a cystic component and solid nodules, similar to other glial tumors like pilocytic astrocytomas and pleomorphic xanthoastrocytomas.^10^ These cystic structures can lead to a mass effect, as seen in this case, and may cause adjacent ventricular compression or midline shift.^5^ Despite these similarities, certain distinguishing features, such as the presence of perivascular rosettes, true and pseudo rosettes, and specific immunohistochemical markers (such as GFAP, EMA, and D240), are critical in identifying ependymomas.^4^

The case highlights that intraparenchymal ependymomas, though rare, may be misdiagnosed as more common tumors such as gliomas or astrocytomas, especially when they lack a clear connection to the ventricular system.^4^ Although the majority of ependymomas are found within the ventricular system, particularly in the fourth ventricle, those located intraparenchymally can present with atypical features, further complicating the diagnosis.^10^ This is especially true for cases with minimal or no direct connection to the ventricular epithelium, making the differential diagnosis even more challenging.^3^

Based on the WHO classification, the anatomical site, histopathological features, and molecular characteristics collectively aid in distinguishing different types of ependymomas. Histologically, ependymomas are defined by the presence of rosettes, including perivascular pseudorosettes and true rosettes around fibrillary matrices.^1^ These rosetting patterns are a hallmark of ependymomas and help differentiate them from other tumors such as gliomas and pilocytic astrocytomas, which typically do not exhibit this rosetting arrangement.^8^ Immunohistochemical staining is crucial for confirming the diagnosis, with ependymomas typically showing positivity for markers such as GFAP (glial fibrillary acidic protein), EMA (epithelial membrane antigen), and D240 (a marker for ependymal differentiation). Negative staining for markers like OLIG-2 and STAT6 further helps rule out other glial tumors such as oligodendrogliomas or other neuronal tumors, confirming the glial origin of the tumor and supporting the diagnosis of ependymoma.^8^

Surgical excision remains the treatment of choice for symptomatic ependymomas, and the extent of resection plays a crucial role in prognosis.^2^ Gross total resection (GTR) is associated with favorable outcomes, with recurrence being relatively rare but possible, particularly in Grade II tumors.^1^ Recurrence is more common in tumors with high Ki-67 proliferation indices, which may necessitate further surgery. The patient in our case had a successful GTR of the parietal lobe lesion with no immediate postoperative problems. However, recurrence occurred after three months of follow-up, necessitating a second operation and postoperative radiotherapy. In correlation with the study by Roberta Rudà in 2022, GTR presents with almost half the likelihood of developing recurrences as compared to subtotal resection (STR).^2^ The approach employed by us in this case was in accordance with current literature and ensures better outcomes.^1^ Regardless, long-term follow-up with imaging is essential.

The role of radiotherapy in the management of ependymomas, particularly recurrent or residual tumors, remains controversial. Some studies report positive outcomes with radiotherapy, especially for patients with recurrent tumors that were not amenable to complete resection.^6^ In contrast, other analyses, such as the Surveillance, Epidemiology, and End Results (SEER) database, suggest the lack of significant improvement with radiotherapy when compared to surgery alone, raising concerns about its importance.^8^ This creates a dilemma in treatment planning. A potential resolution to this conflict lies in individualizing treatment based on tumor grade, extent of resection, and molecular markers. Advanced molecular profiling may help identify patients who are more likely to benefit from radiotherapy, ensuring that its application is targeted rather than generalized.

Recent advances in molecular and genetic research into ependymomas have identified potential therapeutic targets that could pave the way for more targeted treatments in the future.^1^ These include agents that inhibit specific proteins or signaling pathways, such as topoisomerase inhibitors or inhibitors of HIF-1α and STAT3. While these therapeutic options are still in the experimental stages, they have the potential for improving treatment outcomes for patients with recurrent or inoperable ependymomas.^2^

## CONCLUSION

Intraparenchymal ependymomas are rare but should not be overlooked in pediatric patients with atypical neurological symptoms. Accurate diagnosis relies on imaging, histopathology, and immunohistochemistry. While complete surgical resection offers a favorable prognosis, long-term follow-up is crucial due to the risk of recurrence. Emerging molecular therapies hold the key to increasing the safety and efficacy of treatment strategies.

### Authors’ Contributions:

**AK:** Concept and design of study, data acquisition and critical review of the manuscript.

**HW, MFK, HMQ:** Data acquisition, analysis and interpretation and drafted the manuscript.

**HZ:** Concept and design of study, critical review of the manuscript and supervision.

All authors have approved the final version to be published and agreement to be accountable for all aspects of the work in ensuring that questions related to the accuracy or integrity of any part of the work are appropriately investigated and resolved.
